# Group dialectical behaviour therapy for adolescents with emotional dysregulation and maladaptive coping: pilot implementation in Qatar

**DOI:** 10.1192/bji.2024.18

**Published:** 2024-11

**Authors:** Yasser Saeed Khan, Sruthi Mathew, May Jasem AlMeraisi, Majid Alabdulla

**Affiliations:** 1 Medical Lead and Senior Consultant Psychiatrist, Child and Adolescent Mental Health Service, Hamad Medical Corporation, Doha, Qatar; and Clinical Associate Professor, College of Medicine, Qatar University, Doha, Qatar; 2 Clinical Psychologist, Child and Adolescent Mental Health Service, Hamad Medical Corporation, Doha, Qatar; 3 Clinical Director, Child and Adolescent Mental Health Service, Hamad Medical Corporation, Doha, Qatar; 4 Chairman and Senior Consultant Psychiatrist, Mental Health Services, Hamad Medical Corporation, Doha, Qatar; and Psychiatry Clerkship Director and Clinical Associate Professor, College of Medicine, Qatar University, Doha, Qatar

**Keywords:** Mental health services, community mental health teams, personality disorders, self-harm, stigma and discrimination

## Abstract

This paper presents a pioneering pilot implementation of group dialectical behaviour therapy (DBT) for adolescents with maladaptive coping in Qatar's child and adolescent mental health services. The project highlights the positive effect on patient satisfaction and the potential for early intervention with adolescents displaying emotional dysregulation. This pioneering initiative was consistent with local cultural values, stressing the importance of interconnectedness in mental health interventions. The impact of the initiative stresses its significance in diverse cultural contexts, urging further adoption regionally for improved mental health outcomes, particularly among adolescents displaying features of an emerging emotionally unstable personality disorder.

It is estimated that almost 75% of all mental disorders manifest for the first time between the ages of 15 and 25.^1^ It has also been reported that up to 20% of young people will experience an anxiety disorder or a depressive episode by the age of 18 years.^2^ Furthermore, the lifetime prevalence rates of non-suicidal self-injury (NSSI – the destruction of bodily tissue without suicidal intent)^3^ among non-clinical samples of adolescents have been estimated to be 17.2%, compared with 5.5% in adults.^4^ The prevalence of NSSI among adolescents varies, from between 14 and 40% in community populations and 38 and 67% in psychiatric in-patient samples, making it a significant concern among youth.^5^ Suicidal ideation has been reported by 24.66% of adolescents aged 13–19 years.^6^

One factor that has been demonstrated in a growing literature to be associated with high prevalence rates of mental health issues in adolescents is impairment in emotion regulation.^7^ Emotion regulation is the ability to recognise, assess, modify and manage emotions in a socially acceptable way in order to gain and maintain control over strong emotions and acquire adaptive functioning.^8^ Emotional dysregulation, on the other hand, refers to the maladaptive processing of stimuli, external and/or internal, when emotion regulation strategies are impaired.^8^ Emotional dysregulation is mainly characterised by hyperarousal, mood instability, irritability, aggression and maladaptive coping behaviours. Individuals who experience emotional dysregulation during adolescence are more likely to be diagnosed with anxiety disorders, mood disorders and disruptive behaviour disorders in adult life.^9^ Adolescents experiencing high levels of emotional dysregulation have also been found to have elevated rates of self-harm and suicidal behaviour.^10^ One example in this regard is adolescents with emerging borderline personality disorder (BPD). Theories on the development of BPD emphasise that problems with emotion regulation are a core underlying feature of BPD.^11^ The urgent development of programmes aimed at early intervention in emotional dysregulation is therefore crucial for the prevention of severe and persistent mental disorders in the future.^12^

Dialectical behaviour therapy (DBT) is an evidence-based structured intervention originally developed for adults with emotion and behaviour dysregulation and later adapted for adolescents.^13^ It emphasises the ability to effectively manage conflicting and intense emotions. DBT is also effective in increasing coping skills across transdiagnostic populations, including those with anxiety disorders and mood disorders.^14^

In this paper, we describe a pilot implementation of a DBT group for adolescents attending a busy child and adolescent mental health service (CAMHS) in the state of Qatar. Hamad Medical Corporation (HMC) is the largest public healthcare provider in Qatar, and its specialist CAMHS is a typical community-based multidisciplinary team offering specialised support to children and young people below the age of 18 years with a range of mental and behavioural disorders, and their families.^15^ The increasing demand for mental health services at HMC CAMHS, with approximately 1300 referrals in 2022 and 1700 in 2023, alongside workforce challenges, highlights the necessity for effective interventions. Group therapies thus present a strategic approach to enhance service delivery.

The aim of this paper is to illustrate how group DBT can offer suitable support for patients while also optimising resource utilisation in mental health services. The process of this pilot project, one of its kind in the region, along with its impact on young people and their families and future directions are also discussed. To our knowledge, this is the first programme of its kind in the Middle Eastern and North Africa (MENA) region. Since this service evaluation initiative did not involve gathering any new data or recruitment of participants, and was instead a retrospective review of feedback questionnaires, the local Institutional Review Board (IRB) did not deem it necessary to require formal approval. However, departmental approval was obtained to publish relevant data, as advised by the IRB. Consent to publish the direct quotes was not required as the data were all anonymised with no information to identify patients.

## Project structure

Participants were referred to the group by psychiatrists within HMC CAMHS, with a focus on individuals demonstrating severe maladaptive behaviours and emotional dysregulation significantly affecting their daily functioning. In addition, participants were required to be between 12 and 18 years old. Exclusion criteria primarily focused on individuals with active suicidality, to ensure the safety and well-being of all participants. These eligibility criteria ensured the inclusion of adolescents most in need of targeted intervention and support within the group therapy framework.

The group intervention was led by two CAMHS psychologists, together with a primary facilitator and a secondary facilitator. They were supported by CAMHS psychiatric nurses and junior psychology colleagues. On launch, the first group included four adolescents (5 August to 9 December 2020). Subsequent groups comprised nine adolescents (17 March to 14 July 2021) and six adolescents (2 March to 27 April 2022). Out of the 19 adolescents initially enrolled, 4 (from the second group) opted for individual (rather than group) DBT skills training after initiation of the intervention. The first two DBT groups spanned 4 months (16 weekly sessions), accompanied by individual therapy, whereas the last group had to be cut short to 8 weeks, owing to a surge in COVID-19 and holidays. The group modules, including pre-treatment skills, mindfulness, distress tolerance, interpersonal effectiveness and emotion regulation, were aligned with the DBT skills manual for adolescents authored by Rathus & Miller,^16^ ensuring consistency with established therapeutic approaches.

## Impact on service delivery and patient satisfaction

The flexible nature of the group sessions, with variable durations and accommodating external factors, such as the COVID-19 pandemic and holidays, has helped with adapting to the evolving needs of our patient population. This adaptability ensures that the therapeutic intervention remains relevant and accessible despite external challenges. The participating adolescents were asked to complete a feedback form at the end of the course of the group intervention (Supplementary Appendix A, available at https://doi.org/10.1192/bji.2024.18). They were mainly asked to rate the usefulness of the group and whether there had been an improvement in their quality of life since the commencement of the group therapy.

**Fig. 1:**
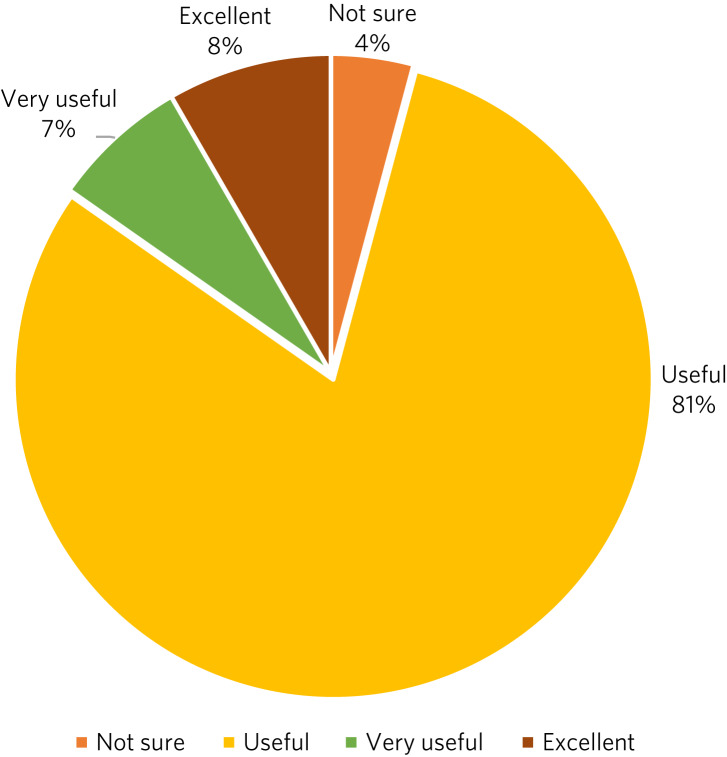
Responses (*n* = 198) from the 15 adolescent participants in group dialectical behaviour therapy in answer to the question ‘How would you rate the usefulness of the group?’.

**Fig. 2:**
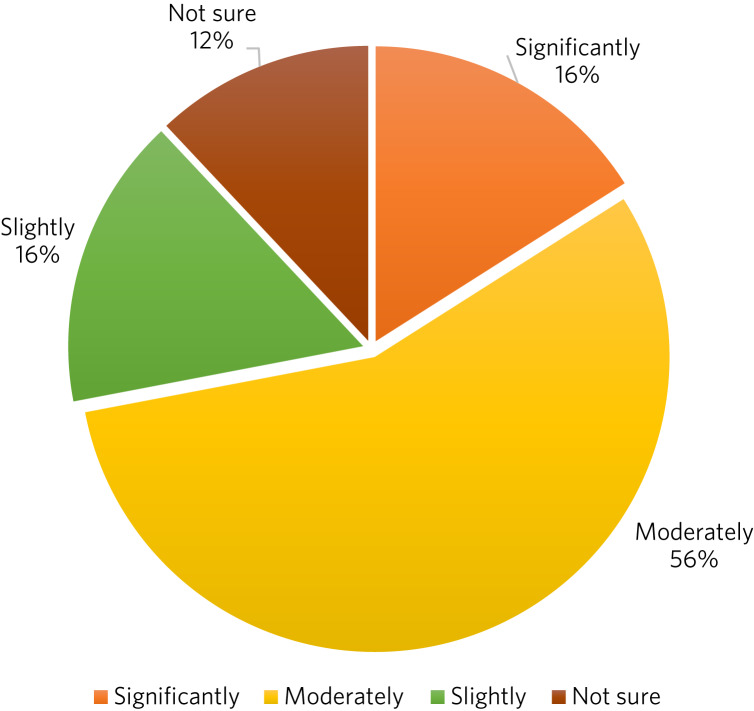
Responses (*n* = 198) from the 15 adolescent participants in group dialectical behaviour therapy (DBT) in answer to the question ‘Do you feel your quality of life improved since participating in the DBT group?’.

A total of 198 responses were received from the participating 15 adolescents for the sessions attended. All responses except 8 (96%) rated the group sessions as either ‘excellent’, ‘very useful’ or ‘useful’ (Fig. 1). Concerning whether there had been an improvement in the quality of life since attending the group sessions, 174 responses out of the total 198 (88%) reported either significant, moderate or slight improvement (Fig. 2). Qualitative feedback data collected from the young people further substantiated the positive impact of group DBT (Box 1). This feedback has been instrumental in understanding the unique experiences and perspectives of the participants.

Box 1 Qualitative feedback from the 15 adolescent participants in group dialectical behaviour therapy

- ‘The group is helpful for people who have less belief in themselves, like me’
- ‘I enjoyed interacting with people and being kind to them while I talked. I liked the role play and the activities’
- ‘All of the activity skills were fun, I found FAST, GIVE, WHAT, and HOW skills very useful’
- ‘It is so nice and calm to come here’
- ‘I use the mindfulness strategies in my class and at home, it has been so helpful’
- ‘Having my own personal self-soothing kit at home and school has helped me better’
- ‘I learned validating myself and others is important’
- ‘I liked the Wise Mind ACCEPTS strategies’
- ‘Very helpful and entertaining to come to the group and learn together’
- ‘I look forward to coming to the group, learning skills and hearing from other girls, it is great that I feel understood’
- ‘I feel calmer than before and I feel I understand people more than before’
- ‘I think my parents have changed maybe because I have also changed’
- ‘I shared these skills with my sister and we practice ACCEPTS and using validation. She reminds me when I forget now’
- ‘Sometimes I forget to use skills, but when I remember I try to think of not judging the experience’
- ‘I taught my close friends when to say NO and FAST skills, we are having fun and they like me for it’
- ‘I don't push my emotions away, and I spend time to understand it now and I know people can't change and it is fine’
- ‘Me and my mom spent last week out by ourselves after a long time and it was really fun, it's a good feeling now with her’
- ‘I usually pause before I shout now and feel angry and I tell myself “it's okay and I can deal with this”’
- ‘I have started my scrapbook and my reading, it has helped me Distract’
- ‘This skills group has been so useful for all of us and we know we can share experiences here and not feel alone’

Specific instances of positive feedback shown in Box 1 highlight improved coping mechanisms, increased emotion regulation and strengthened interpersonal skills reported by participants. The sense of community fostered in group sessions was particularly praised, with participants expressing a feeling of shared understanding and support. In addition to the feedback provided by the participating adolescents, the improvement in the participants’ well-being and therapeutic progress was further supported by the accounts of their parents and treating psychologists, providing objective evidence of the intervention's usefulness.

## Discussion

Group dialectical behaviour therapy (DBT) has had a positive effect on the delivery of healthcare services to adolescents in a Qatar CAMHS and has contributed to increased satisfaction among patients and their families. This novel approach has proven to be a valuable addition to our existing services. The combination of group therapy with individual sessions has created a comprehensive treatment framework. The structured modules, including pre-treatment skills, mindfulness, distress tolerance, interpersonal effectiveness and emotion regulation, provide a holistic approach, to address maladaptive coping strategies among adolescents. The structure has allowed for a more focused understanding of individual needs, as group dynamics often reveal additional insights that may not be as apparent in individual sessions.

The project serves to reiterate the importance of the growing literature emphasising the role of emotional dysregulation in adolescent psychopathology. Group DBT proved productive in addressing emotional dysregulation, as evidenced by the feedback provided by the young people and their parents. Recognising the importance of addressing emotional dysregulation in adolescence, this initiative contributes to preventing the escalation of mental health issues to severe and persistent disorders. The exchange of perspectives and insights among participants can be of significant mutual benefit. Adolescents experiencing maladaptive coping often feel isolated in their struggles. Group DBT breaks down these barriers by creating a space where individuals can relate to one another's experiences, reducing the stigma associated with emotional dysregulation. Group DBT, as demonstrated in this pilot, not only provides an understanding and supportive environment for adolescents but also incorporates the views of their families in the therapeutic process.

The implementation of group dialectical DBT for adolescents in Qatar has regional significance. The Middle East faces the challenge of addressing mental health stigma, making it crucial to promote accessible and culture-sensitive interventions while adopting a non-judgemental approach.^17^ This pilot implementation demonstrates how evidence-based practices can be introduced effectively in diverse cultural contexts. This programme acknowledged the importance of family involvement and community support and was therefore consistent with cultural expectations. The communal nature of group therapy aligned with local cultural values and stressed the importance of interconnectedness and shared experiences. Such an approach is deemed integral to the administering of mental health interventions in the MENA region.

## Conclusion

- This pioneering pilot implementation in a Qatar CAMHS demonstrates the feasibility and significance of group DBT for adolescents with emotion regulation challenges and maladaptive coping.
- The positive outcome highlights the importance of early intervention in emotional dysregulation, potentially preventing the emergence of severe and persistent mental disorders, including personality disorders.
- There is an urgent need for original research to understand the role of group DBT in the management of emotional dysregulation to advance mental health initiatives in the MENA region.
- The utilisation of experiences from this project can help regional services to identify areas for improvement and potentially encourage other services to implement similar initiatives.

## Supporting information

Khan et al. supplementary material Khan et al. supplementary material

## Data Availability

The data that support the findings of this study are available on request from the corresponding author.

## References

[1] Dooley BA, Fitzgerald A. My World Survey: National Study of Youth Mental Health in Ireland. Headstrong and UCD School of Psychology, 2012.

[2] Werner-Seidler A, Perry Y, Calear AL, Newby JM, Christensen H. School-based depression and anxiety prevention programs for young people: a systematic review and meta-analysis. Clin Psychol Rev 2017; 51: 30–47. 27821267 10.1016/j.cpr.2016.10.005

[3] Halicka J, Kiejna A. Non-suicidal self-injury (NSSI) and suicidal: criteria differentiation. Adv Clin Exp Med 2018; 27: 257–61. 29521070 10.17219/acem/66353

[4] Swannell SV, Martin GE, Page A, Hasking P, St John NJ. Prevalence of nonsuicidal self-injury in nonclinical samples: systematic review, meta-analysis and meta-regression. Suicide Life Threat Behav 2014; 44: 273–303. 24422986 10.1111/sltb.12070

[5] Cloutier P, Martin J, Kennedy A, Nixon MK, Muehlenkamp JJ. Characteristics and co-occurrence of adolescent non-suicidal self-injury and suicidal behaviours in pediatric emergency crisis services. J Youth Adolesc 2010; 39: 259–69. 19856090 10.1007/s10964-009-9465-1

[6] Zygo M, Pawłowska B, Potembska E, Dreher P, Kapka-Skrzypczak L. Prevalence and selected risk factors of suicidal ideation, suicidal tendencies and suicide attempts in young people aged 13–19 years. Ann Agricult Environ Med 2019; 26: 329–36. 10.26444/aaem/93817 31232067

[7] Pat-Horenczyk R, Cohen S, Ziv Y, Achituv M, Asulin-Peretz L, Blanchard TR, et al Emotion regulation in mothers and young children faced with trauma. Infant Ment Health J 2015; 36: 337–48. 25941026 10.1002/imhj.21515

[8] Paulus FW, Ohmann S, Möhler E, Plener P, Popow C. Emotional dysregulation in children and adolescents with psychiatric disorders. a narrative review. Front Psychiatry 2021; 12: 628252. 34759846 10.3389/fpsyt.2021.628252 PMC8573252

[9] Althoff RR, Verhulst FC, Rettew DC, Hudziak JJ, van der Ende J. Adult outcomes of childhood dysregulation: a 14-year follow-up study. J Am Acad Child Adolesc Psychiatry 2010; 49: 1105–16. 20970698 10.1016/j.jaac.2010.08.006 PMC2965164

[10] Chesney E, Goodwin GM, Fazel S. Risks of all-cause and suicide mortality in mental disorders: a meta-review. World Psychiatry 2014; 13: 153–60. 24890068 10.1002/wps.20128 PMC4102288

[11] Crowell SE, Beauchaine TP, Linehan MM. A biosocial developmental model of borderline personality: elaborating and extending Linehan's theory. Psychol Bull 2009; 135: 495–510. 19379027 10.1037/a0015616 PMC2696274

[12] Winsper C, Marwaha S, Lereya ST, Thompson A, Eyden J, Singh SP. Clinical and psychosocial outcomes of borderline personality disorder in childhood and adolescence: a systematic review. Psychol Med 2015; 45: 2237–51. 25800970 10.1017/S0033291715000318

[13] Miller AL, Rathus JH, Linehan MM. Dialectical Behavior Therapy with Suicidal Adolescents. Guilford Press, 2006.

[14] Neacsiu AD, Eberle JW, Kramer R, Wiesmann T, Linehan MM. Dialectical behavior therapy skills for transdiagnostic emotion dysregulation: a pilot randomized controlled trial. Behav Res Ther 2014; 59: 40–51. 24974307 10.1016/j.brat.2014.05.005

[15] Khan YS, Al-Shamlawi M, Phiri L, Alabdulla M. Triage of referrals in a child and adolescent mental health service in Qatar: reducing waiting times and promoting needs-based prioritisation. BJPsych Int 2021; 18: 67–70. 34382959 10.1192/bji.2021.10 PMC8314989

[16] Rathus JH, Miller AL. DBT Skills Manual for Adolescents. Guilford Publications, 2014.

[17] Khatib HE, Alyafei A, Shaikh M. Understanding experiences of mental health help-seeking in Arab populations around the world: a systematic review and narrative synthesis. BMC Psychiatry 2023; 23(1): 324. 37161342 10.1186/s12888-023-04827-4 PMC10170733

