# Supplementary material for: Group dialectical behaviour therapy for adolescents with emotional dysregulation and maladaptive coping: pilot implementation in Qatar
Source: BJPsych Int. 2024 Nov;21(4):83–7. doi: 10.1192/bji.2024.18 (PMC12022851; doi:10.1192/bji.2024.18)
Supplement: Khan et al. supplementary material [file S2056474024000187sup001.docx]

Appendix A

**Child and Adolescent Mental Health Services (CAMHS)**

**Psychology Services**

DBT Group Therapy

Adolescent feedback form

Date : ____________ Week : _________________

Thank you for attending the DBT group, the feedback form is a way to improve the group support. Please note the data entered here is confidential. Kindly fill the feedback form.

1. How would you rate the usefulness of the group?

| Poor | Not useful | Useful | Very useful | Excellent |
| --- | --- | --- | --- | --- |

|  |
| --- |

1. What skills from the group did you find beneficial?
2. Do you feel your quality of life improved since participating in DBT group?

- Yes, significantly
- Yes, moderately
- Yes, slightly
- No difference
- Not at all
- Not sure

#### What do you like about the group session today?

|  |
| --- |

#### What changes or recommendations would you have for group?

|  |
| --- |

1. Please add any additional remarks or suggestion

|  |
| --- |

Thank you for your feedback 😊
